# Pirfenidone normalizes the tumor microenvironment to improve chemotherapy

**DOI:** 10.18632/oncotarget.15534

**Published:** 2017-02-20

**Authors:** Christiana Polydorou, Fotios Mpekris, Panagiotis Papageorgis, Chrysovalantis Voutouri, Triantafyllos Stylianopoulos

**Affiliations:** ^1^ Cancer Biophysics Laboratory, Department of Mechanical and Manufacturing Engineering, University of Cyprus, Nicosia, Cyprus; ^2^ Department of Life Sciences, Program in Biological Sciences, European University Cyprus, Nicosia, Cyprus

**Keywords:** tumor perfusion, vessel compression, breast cancer, drug delivery, biomechanics

## Abstract

Normalization of the tumor microenvironment by selectively targeting components of the tumor extracellular matrix has been recently proposed to have the potential to decompress tumor blood vessels, increase vessel perfusion and thus, improve drug delivery and the efficacy of cancer therapy. Therefore, we now need to identify safe and well tolerated pharmaceutical agents that are able to remodel the microenvironment of solid tumors and enhance chemotherapy. In this study, we repurposed Pirfenidone, a clinically approved anti-fibrotic drug for the treatment of idiopathic pulmonary fibrosis, to investigate its possible role on tumor microenvironment normalization. Using two orthotopic mammary tumor models we demonstrate that Pirfenidone reduces collagen and hyaluronan levels and, as a result, significantly increases blood vessel functionality and perfusion and improves the anti-tumor efficacy of doxorubicin. Reduction of extracellular matrix components were mediated via TGFβ signaling pathway inhibition due to downregulation of TGFβ1, COL1A1, COL3A1, HAS2, HAS3 expression levels. Our findings provide evidence that repurposing Pirfenidone could be used as a promising strategy to enhance drug delivery to solid tumors by normalizing the tumor microenvironment.

## INTRODUCTION

Current chemotherapeutic agents, in sufficient concentrations, are potent enough to kill cancer cells. Nonetheless, failure of standard chemotherapies for the treatment of desmoplastic cancers (e.g. breast and pancreatic cancers) is in large part due to the fact that these drugs are unable to reach cancer cells in amounts sufficient to cause complete cure [1]. In these tumors, blood vessels are compressed and sometimes collapsed, drastically reducing perfusion and resulting in insufficient delivery of any blood-borne therapeutic agent [2–4]. Desmoplastic tumors are characterized by an abnormally dense extracellular matrix (ECM) and vessel compression is an effect of mechanical forces developed within the tumor due to uncontrolled cancer cell proliferation that strains the ECM of the tumor microenvironment [5, 6].

Normalization of the abnormal tumor microenvironment by selectively targeting components of the tumor ECM has been proposed to have the potential to alleviate mechanical forces, decompress tumor vessels and improve perfusion [7–10]. As a result drug delivery and therapeutic outcomes are improved. Specifically, it was shown that treatment with PEGPH20, a PEGylated human recombinant hyaluronidase, decreased hyaluronan levels in pancreatic ductal adenocarcinoma tumors *in vivo*, causing an increase in vessel diameter and functional vessel density and thus, resulting in the improvement in drug delivery and treatment efficacy [11, 12]. Moreover, depletion of collagen and/or hyaluronan by repurposing the angiotensin receptor blocker losartan (Cozaar^®^, Merck & Co., Inc), or using the monoclonal antibody 1D11, an inhibitor of TGF-β signaling pathway, improved the delivery of therapeutics *in vivo* by inducing blood vessel decompression and improving perfusion in a variety of breast and pancreatic tumors in mice [2, 13, 14]. From the above mentioned agents, only losartan is clinically approved, as an anti-hypertensive drug, and based on preclinical findings [2], a phase II trial using losartan and FOLFIRINOX has been initiated for the treatment of pancreatic ductal adenocarcinoma (PDACs) patients (clinicaltrials.org identifier NCT01821729). Additionally, a phase III trial of PEGPH20 combined with nab-paclitaxel plus gemcitabine has been recently started for patients suffering from PDACs (clinicaltrials.org identifier NCT02715804). Given the promise of such combinatorial treatments to improve cancer therapy, we need to identify clinically approved pharmaceutical agents, other than losartan, that can be used in order to improve the efficacy of chemotherapeutic drugs and may be added to cancer patients’ treatment regimen. In this notion, we hypothesized that Pirfenidone, an anti-fibrotic drug could be repurposed to alleviate mechanical forces in solid tumors and thus, improve delivery and efficacy of common anti-cancer drugs.

Pirfenidone [5-methyl-1-2-[1H]-pyridone] is an anti-fibrotic drug, commonly used for the treatment of idiopathic pulmonary fibrosis. It was first approved in Japan under the trade name Pirespa^®^ and some years later in Europe, United States and Canada under the trade name Esbriet^®^ (Roche Pharmaceuticals) for the same cure. A few studies using either *in vitro* experiments or animal models have shown that Pirfenidone inhibits fibrosis by downregulation of the TGFβ signaling pathway and collagen synthesis [15–17]. However, to date, no studies have analyzed the effects of Pirfenidone on remodeling the tumor ECM to improve the delivery and efficacy of cancer therapeutics. To investigate this, we employed two orthotopic breast cancer mouse models: a xenograft model using the human MCF10CA1a cell line and a syngeneic model using the 4T1 murine mammary adenocarcinoma cell line. We demonstrate that Pirfenidone normalizes the tumor ECM and as a result significantly reduces mechanical forces, decompresses tumor vessels, improves perfusion, and enhances the efficacy of doxorubicin in breast tumors. We further show that this effect is due to the reduction of collagen and hyaluronan tumor components as a result of TGFβ signaling pathway inhibition and downregulation of collagen I (COL1A1), collagen 3 (COL3), hyaluronan synthase 2 (HAS2), hyaluronan synthase 3 (HAS3) and transforming growth factor-beta 1 (TGFβ1) gene expression.

## RESULTS

### Pirfenidone normalizes the tumor microenvironment by reducing collagen and hyaluronan levels in a dose-depended manner

**Figure 1:**
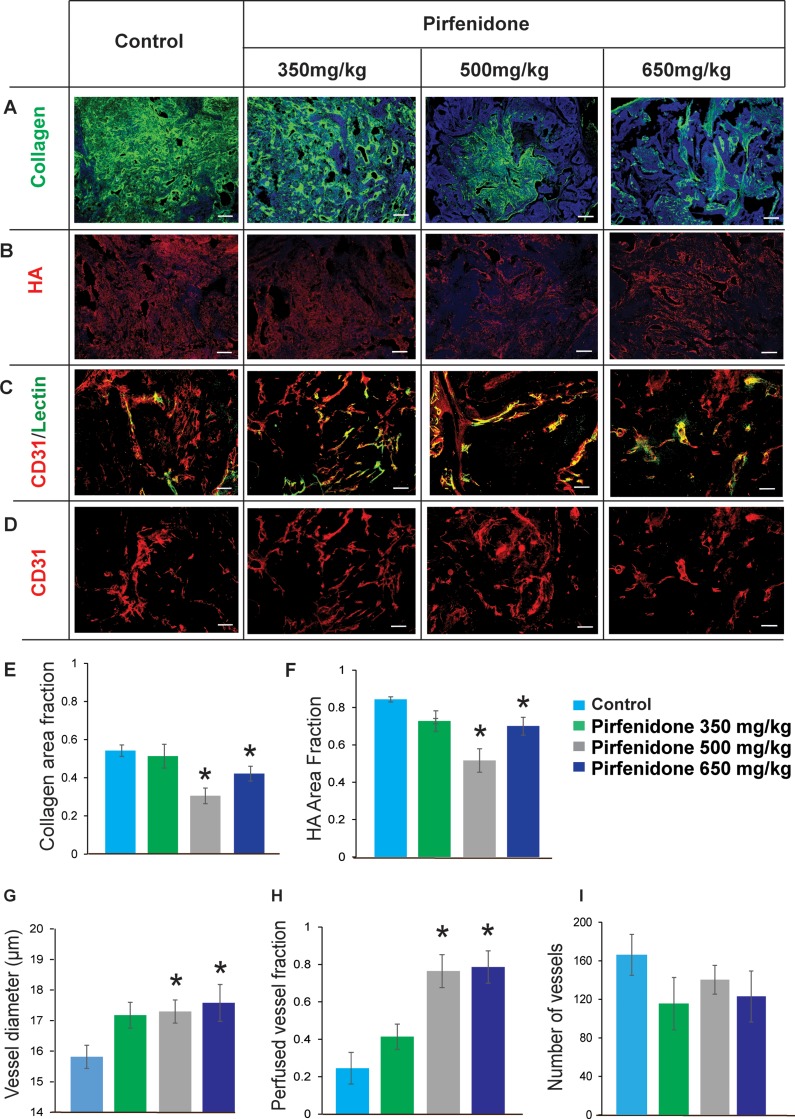
Pirfenidone remodels the tumor microenvironment by reducing collagen and hyaluronan levels in a dose-depended manner (**A**–**D**) Representative immunofluorescent images from whole tumor samples of control and Pirfenidone-treated tumors for collagen, hyaluronan (HA), CD31 and lectin. (**E**, **F**) Area fraction analysis shows that 500 and 650 mg/kg Pirfenidone reduces collagen and hyaluronan levels significantly compared to the control group. (**G**–**I**) The 500 and 650 mg/kg dose of Pirfenidone increased significantly mean vessel diameter, improving fraction of perfused vessels but had no effect on number of tumor blood vessels (*n* = 6–8). Asterisks indicate a statistically significant difference between compared groups (*p* < 0.05). Scale bar: 100 μm.

To investigate the dose-dependent effects of Pirfenidone on tumor ECM, we initially developed an orthotopic xenograft mouse model for breast cancer using the human MCF10CA1a breast cancer cell line. Three groups of mice received Pirfenidone orally on a daily basis at doses of 350, 500 and 600 mg/kg respectively [17], starting 4 days post-implantation of cancer cells in the mammary fat pad for a period of 33 days. First, we observed that treatment with Pirfenidone had no effect on tumor growth compared to control at all doses (Supplementary Figure 1). Histological analysis with immunofluorescence staining of tumor sections showed that while treatment with 350 mg/kg Pirfenidone had no significant effect on ECM tumor content compared to tumors from control mice that received sterile water, increased Pirfenidone concentrations at 500 and 650 mg/kg, significantly decreased collagen and hyaluronan levels (Figure 1A, 1B). Interestingly, we found that this reduction was the greatest for both components at the dose of 500 mg/kg as confirmed by area fraction quantification (Figure 1E, 1F). The area fraction of collagen and hyaluronan for the 500 mg/kg Pirfenidone-treated tumors was reduced by ∼50%, whereas in 650 mg/kg -treated animals it was decreased by ∼30%, compared to control-treated mice. Moreover, fibronectin at 500 mg/kg Pirfenidone-treated tumors was reduced by ∼20% (Supplementary Figure 2). In order to examine the effect of ECM reduction on the functionality of tumor blood vessels, we first calculated the percentage of perfused blood vessels of control and Pirfenidone-treated tumors as well as the vessel diameter. Tumor tissue cryosections from all groups were stained with antibodies against the endothelial marker CD31 and biotinylated lectin, which specifically marks the functional tumor vessels [2] (Figure 1C, 1D). The fraction of perfused vessels was calculated based on the ratio of the number of biotinylated lectin positive (+) to the number of CD31 positive (+) vessels. We measured no significant change in tumor perfusion in samples treated with 350mg/kg Pirfenidone. In contrast, in tumors isolated from mice treated with 500 mg/kg and 600 mg/kg Pirfenidone, we observed a statistically significant increase in perfusion. Interestingly, the fraction of perfused vessels was increased by more than 3-fold in both groups compared to control (Figure 1H). Consistent with these findings, calculation of vessel diameters in these tumors indicated that tumors treated with 500 and 650 mg/kg Pirfenidone had a statistically significant increased vessel diameter, suggesting that improved tumor perfusion is an effect of vessel decompression (Figure 1G). Despite the fact that we observed relatively small increases in vessel diameter, these findings are in agreement with previous studies showing that even small increases in vessel diameter can significantly improve blood flow [2, 5]. Moreover, quantification of CD31 staining between different groups showed that Pirfenidone treatment did not affect the total number of blood vessels (Figure 1I), suggesting that Pirfenidone can improve perfusion without affecting tumor angiogenesis. Furthermore, measurements of the interstitial fluid pressure (IFP) and the elastic modulus of the tumors (Supplementary Figure 3) showed that even though tumors became softer for both 500 and 650 mg/kg Pirfenidone, only for the 500 mg/kg dose, IFP was significantly decreased.

Taken together, these data indicate that the dose of 500 mg/kg Pirfenidone is the optimal to induce normalization of the tumor microenvironment and therefore was selected for the subsequent analyses. The notion that Pirfenidone dose of 500 mg/kg show stronger effects than 650 mg/kg Pirfenidone could be due to nonspecific target effects at higher doses.

### Pirfenidone decreases the levels of ECM components and increases blood vessel functionality in a syngeneic 4T1 breast tumor model

**Figure 2:**
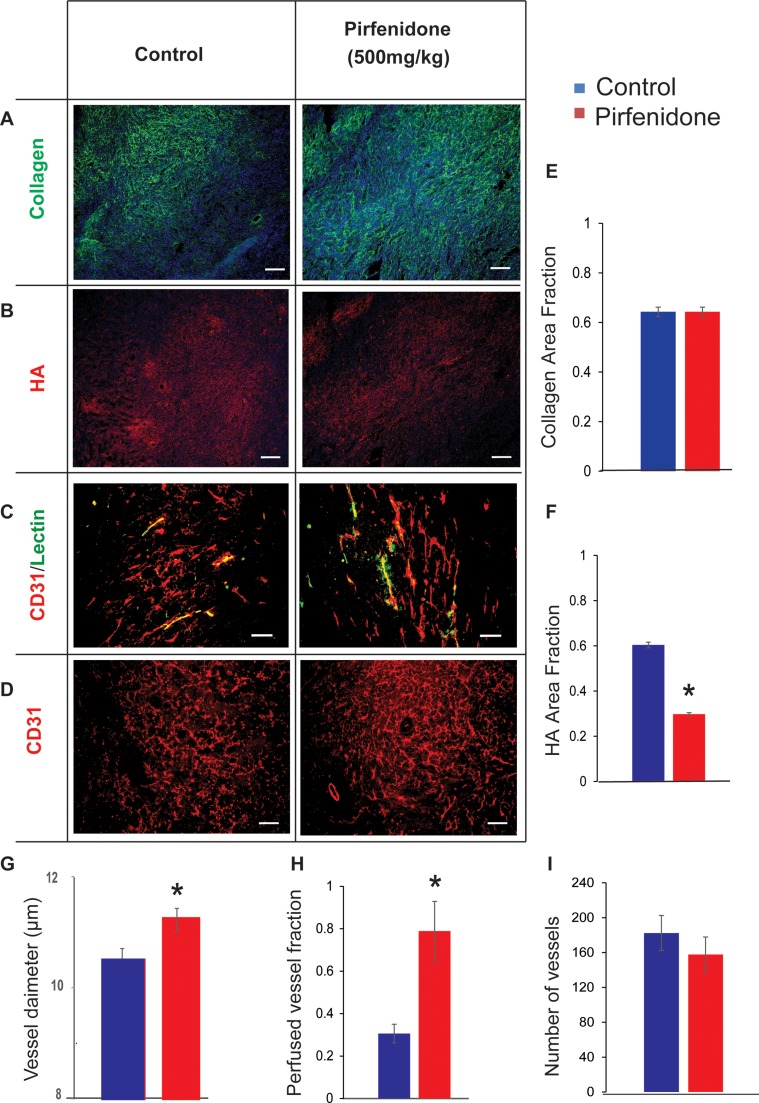
Pirfenidone decreases the levels of ECM components and normalizes blood vessel perfusion in 4T1 breast tumors (**A**–**D**) Representative immunofluorescent images from histological analysis show the effect of Pirfenidone on collagen, hyaluronan (HA), CD31 and lectin. (**E**, **F**) Quantification of area fractions of collagen and hyaluronan indicates that hyaluronan but not collagen levels are significantly reduced compared to control tumors. (**G**–**I**) Quantification of blood vessel diameter, perfused vessels fraction and number of vessels in control and Pirfenidone-treated tumors indicate that Pirfenidone increases significantly blood vessel functionality by decompressing tumor vessels without affecting the number of vessels (*n* = 6–8). Asterisks indicate statistically significant difference between groups (*p* < 0.05). Scale bar: 100 μm.

Next, we wanted to confirm these effective changes on tumor microenvironment and tumor blood vessel functionality caused by Pirfenidone in another orthotopic breast tumor model. For this set of experiments we employed a syngeneic model using mouse 4T1 breast cancer cells implanted in BALB/C mice. Daily administration of 500 mg/kg Pirfenidone caused no significant reduction of collagen content (Figure 2A) but hyaluronan levels were dramatically reduced compared to the control group (Figure 2B). This was confirmed by area fraction quantification for both collagen (Figure 2E) and hyaluronan (Figure 2F). Despite the fact that there was no difference in collagen levels between the control and Pirfenidone-treated tumors, hyaluronan area fraction was reduced by almost 50%. Furthermore, Pirfenidone reduced significantly fibronectin levels (Supplementary Figure 2).

Since there was no effect on collagen in 4T1 tumors, it was interesting to examine the possible action of Pirfenidone on myofibroblast-like cells. We performed immunofluorescence staining for the positive myofibroblast-like cells using the alpha-smooth muscle actin (a-sma) antibody. We found that there was no reduction on its expression in Pirfenidone-treated tumors compared to controls (Supplementary Figure 4) indicating that in our study Pirfenidone is not acting on myofibroblast-like cells. Moreover, in order to examine the effect of ECM reduction on the functionality of tumor blood vessels, we calculated the percentage of perfused blood vessels. Tissue cryosections were stained with antibodies against or CD31 and biotinylated lectin (Figure 2C, 2D). Again, the fraction of perfused vessels was calculated based on the ratio of the number of biotinylated lectin positive (+) to the CD31 positive (+) vessels. Importantly, there was a significant increase in tumor perfusion in Pirfenidone-treated samples compared to controls by 60%, which was associated with decompression of tumor blood vessels (Figure 2G, 2H). Finally, the total area of vessels (CD31+ area) remained unaffected (Figure 2D, 2I), suggesting that Pirfenidone, at the selected dose of 500 mg/kg, did not affect tumor angiogenesis.

### Pirfenidone alleviates solid and fluid stresses in MCF10CA1a and 4T1 breast tumors

**Figure 3:**
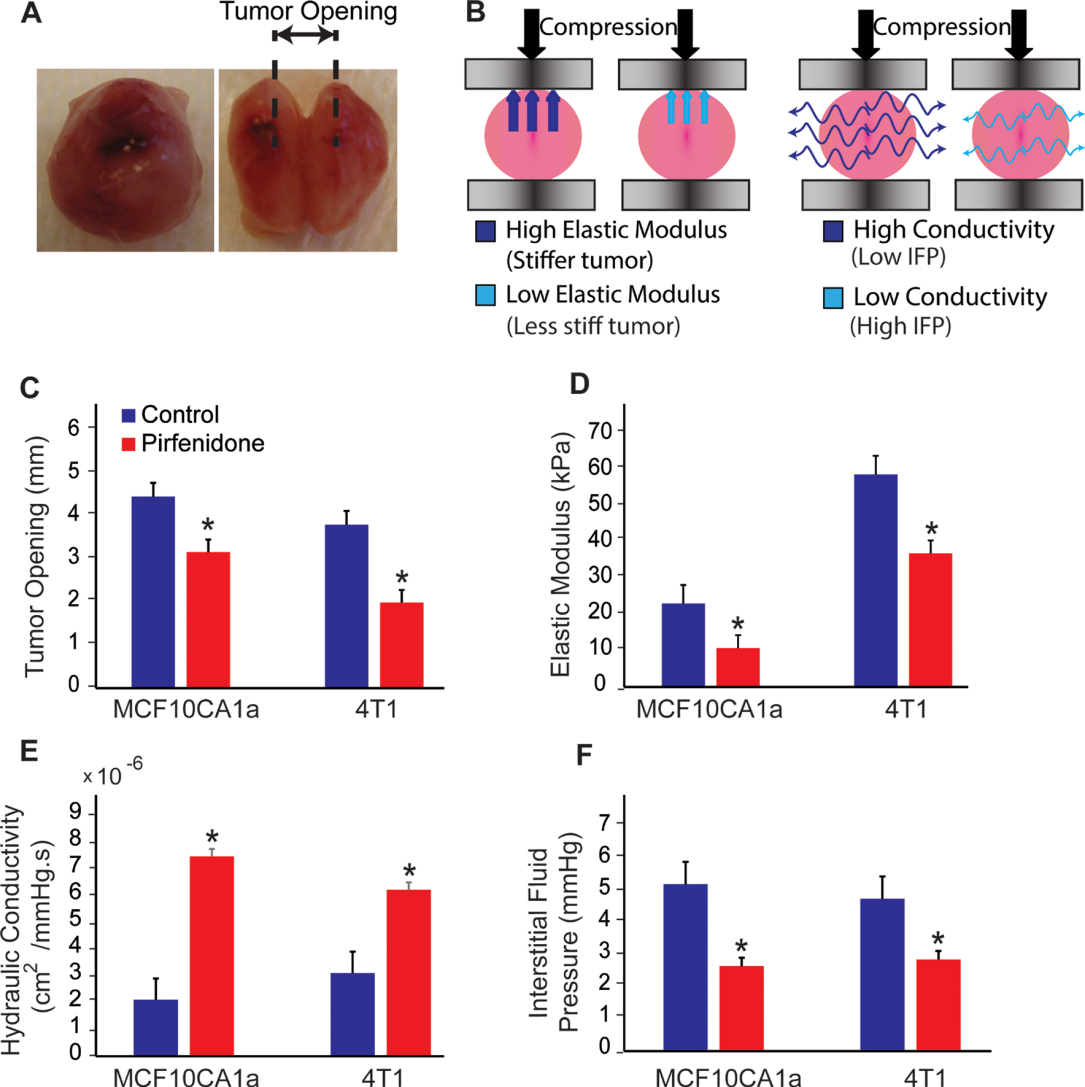
Pirfenidone normalizes solid and fluid stress in tumors (**A**) Representative images from tumor opening experiment indicating that a cut made at ∼80% of tumor thickness along the main axis results in opening of the tumor due to stress release by tissue relaxation. (**B**) Schematic of the unconfined compression set up. Stiffer tumors have a higher elastic modulus and resist stronger to compression. Tumors with larger values of hydraulic conductivity allow easier fluid flow through their mass and thus, more fluid exits the tissue during compression. (**C**) Measurements of tumor opening for the two different tumor models indicate that treatment with 500 mg/kg Pirfenidone leads to lower values of relaxation compared to control and hence to lower levels of solid stress. (**D**) The values of the elastic modulus for the Pirfenidone-treated tumors are shown to be significantly lower in both models. (**E**) Pirfenidone treatment increases the hydraulic conductivity of both tumor types, which in turn causes a decrease in the (**F**) interstitial fluid pressure.

Improved perfusion *via* reduction of the tumor ECM and decompression of tumor blood vessels has been attributed to alleviation of intratumoral solid stresses, i.e., the forces originated from the solid phase of a tumor. Furthermore, delivery of drugs can be improved not only by increasing perfusion, which brings a larger volume of blood carrying the drug into the tumor, but also by alleviation of the IFP which increases the amount of drug that is transferred across the tumor vessel walls from the vascular to the interstitial space of the tumor [18]. Therefore, we carried out experiments to measure whether treatment with Pirfenidone affected solid stress and IFP. For the solid stress measurement, we performed two experiments: we employed the tumor opening experiment [5] to measure the solid stress that remains in the tissue when the tumor is excised and the stress-strain experiment to measure the elastic modulus of the tumors. For the tumor opening experiment, upon excision the tumors were cut along their longest axis at approximately 80% of their thickness and then they were allowed to relax for 10 min. Owing to the solid stresses that remain in the tumor even though it has been removed from the mouse, cutting of the tumor results in the swelling of the interior of the tumor and opening at the periphery. After the tumor opens we measured the distance between the two hemispheres (Figure 3A). Pirfenidone-induced reduction of the ECM decreased tumor opening, which is indicative of alleviation of solid stresses (Figure 3C) and also reduced the elastic modulus of both tumor types, making the tumors less stiff and thus, reducing their capacity to develop high stresses (Figure 3B, 3D). These data suggest that treatment with Pirfenidone decreased solid stresses in both MCF10CA1a and 4T1 tumor models. Moreover, we tested the effect of Pirfenidone on the hydraulic conductivity (Figure 3B, 3E) and IFP (Figure 3F) of the tumors. Hydraulic conductivity describes the ease by which interstitial fluid percolates through the pores of the tumor interstitial space (Figure 3B). Low hydraulic conductivity hinders fluid flow through the pores of the tumor interstitial space, which results in accumulation of interstitial fluid, which in turn raises IFP. For hydraulic conductivity calculations, we performed *ex vivo* stress-relaxation experiments where the tumor sample is placed between two platens, it is compressed and held to a constant compression so that the interstitial fluid will equilibrate and some of it will exit the tissue (Figure 3B and Supplementary Figure 5). The fluid equilibration (or relaxation) time is indicative of the hydraulic conductivity, the faster the tissue equilibrates the higher its conductivity will be. Pirfenidone-treated tumors showed an increased hydraulic conductivity in both preclinical tumor models (Figure 3E). Furthermore, Pirfenidone significantly decreased IFP in both tumor models (Figure 3F). In conclusion, our data show that reduction of ECM composition can alleviate both solid stresses and fluid pressures in tumors to obtain values closer to the (zero) values of normal tissues.

### Pirfenidone enhances systemic administration of doxorubicin to tumors but not to normal tissues

**Figure 4:**
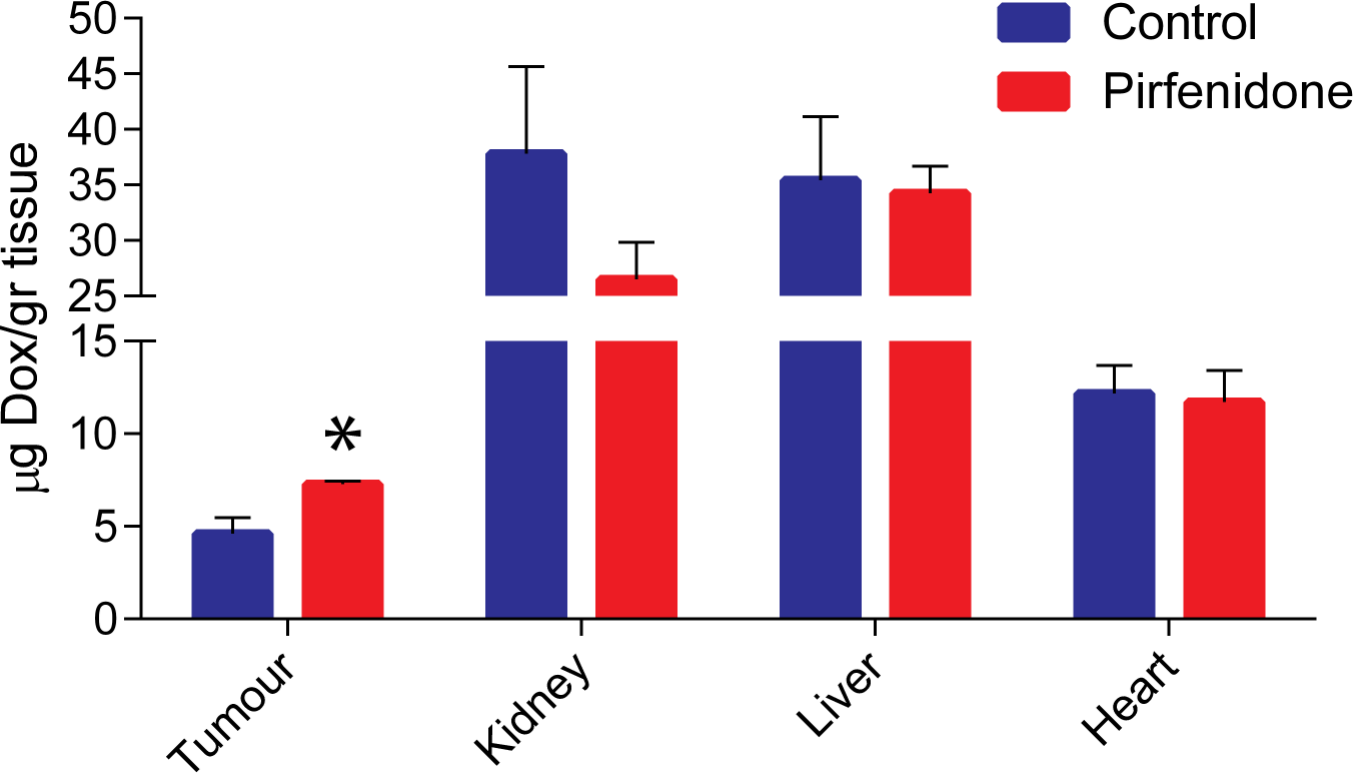
Pirfenidone increases intratumoral delivery of doxorubicin Quantification of doxorubicin concentration in control and Pirfenidone-treated 4T1 breast tumors as well as in kidney, liver and heart tissues. 9 mg/kg doxorubicin was injected intravenously to the animals 4 hours prior to sacrifice. Pirfenidone improves the delivery of doxorubicin in tumors by ∼ 50% without affecting delivery to normal tissues. Asterisk indicates statistically significant differences between compared groups (*p* < 0.05, *n* = 6).

To relate the normalization of the tumor microenvironment to the delivery of chemotherapy, we performed a biodistribution analysis in 4T1 tumors. Specifically, 9 mg/kg of doxorubicin was injected intravenously (i.v.) to animals 4 hours prior to sacrifice. Doxorubicin concentration in tissue sample homogenates was determined by quantification of fluorescence intensity (Ex.: 470 nm, Em: 590 nm) and calculated based on a standard curve generated by addition and measurement of known amounts of doxorubicin to normal tissue homogenates of non-treated animals. A significant increase in the delivery of doxorubicin in Pirfenidone-treated tumors, by approximately 50%, was observed compared to the control group without causing any effects to the delivery of the drug to normal tissues (Figure 4).

### Pirfenidone increases anti-tumor efficacy of chemotherapy

**Figure 5:**
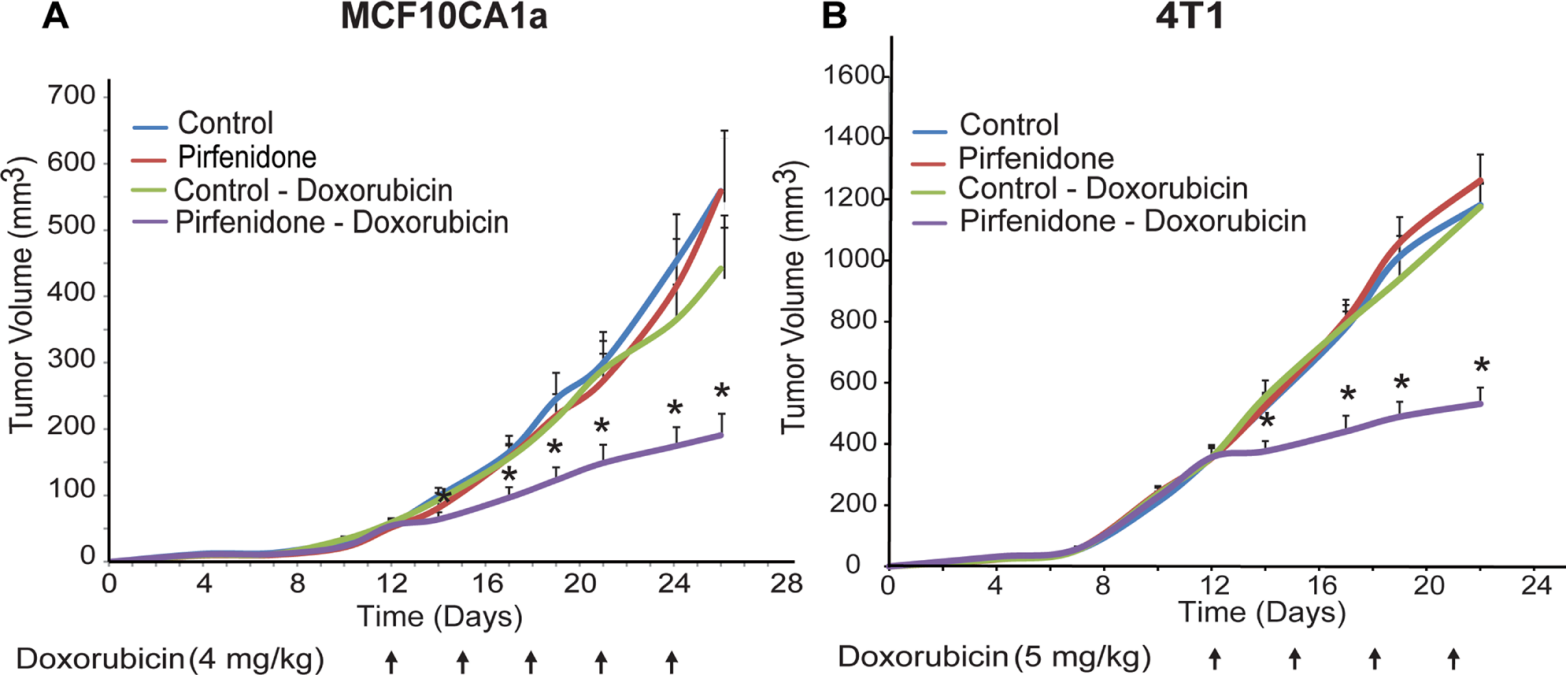
Pirfenidone increases anti-tumor efficacy of chemotherapy Tumor volume growth rates of (**A**) orthotopic MCF10CA1a human breast tumors and (**B**) 4T1 murine breast tumors implanted in female CD1 nude or BALB/c mice, respectively. Control, 500 mg/kg Pirfenidone or doxorubicin alone treated-animals had no effect on tumor growth in both tumor models. Combination treatment of 500 mg/kg Pirfenidone and 4 mg/kg doxorubicin for the MCF10CA1a or 5 mg/kg for the 4T1 tumors showed a significant reduction in tumor growth (*n* = 8–10). The error bars denote the standard error of the mean. Asterisks indicate a statistically significant difference between compared groups (*p* < 0.05).

To correlate enhanced delivery of chemotherapy to treatment efficacy, we performed tumor growth studies using both the MCF10CA1a and 4T1 tumor models. In these studies, doxorubicin and Pirfenidone were administered either alone or in combination and the tumor volume was measured throughout the treatment period. Administration of 500 mg/kg Pirfenidone in both MCF10CA1a and 4T1 tumor models did not influence primary tumour growth. Similarly, 4 mg/kg doxorubicin, for MCF10CA1a tumors, or 5 mg/kg doxorubicin for the 4T1 tumors, (i.p.) had no effect on tumor growth in either animal model. However, combinatorial treatment using both Pirfenidone and doxorubicin resulted in a significant delay in tumor growth in both models (Figure 5). Therefore, Pirfenidone-induced normalization of tumor ECM can improve the efficacy of chemotherapy.

### Pirfenidone downregulates the expression of genes involved in remodeling of the ECM

**Figure 6:**
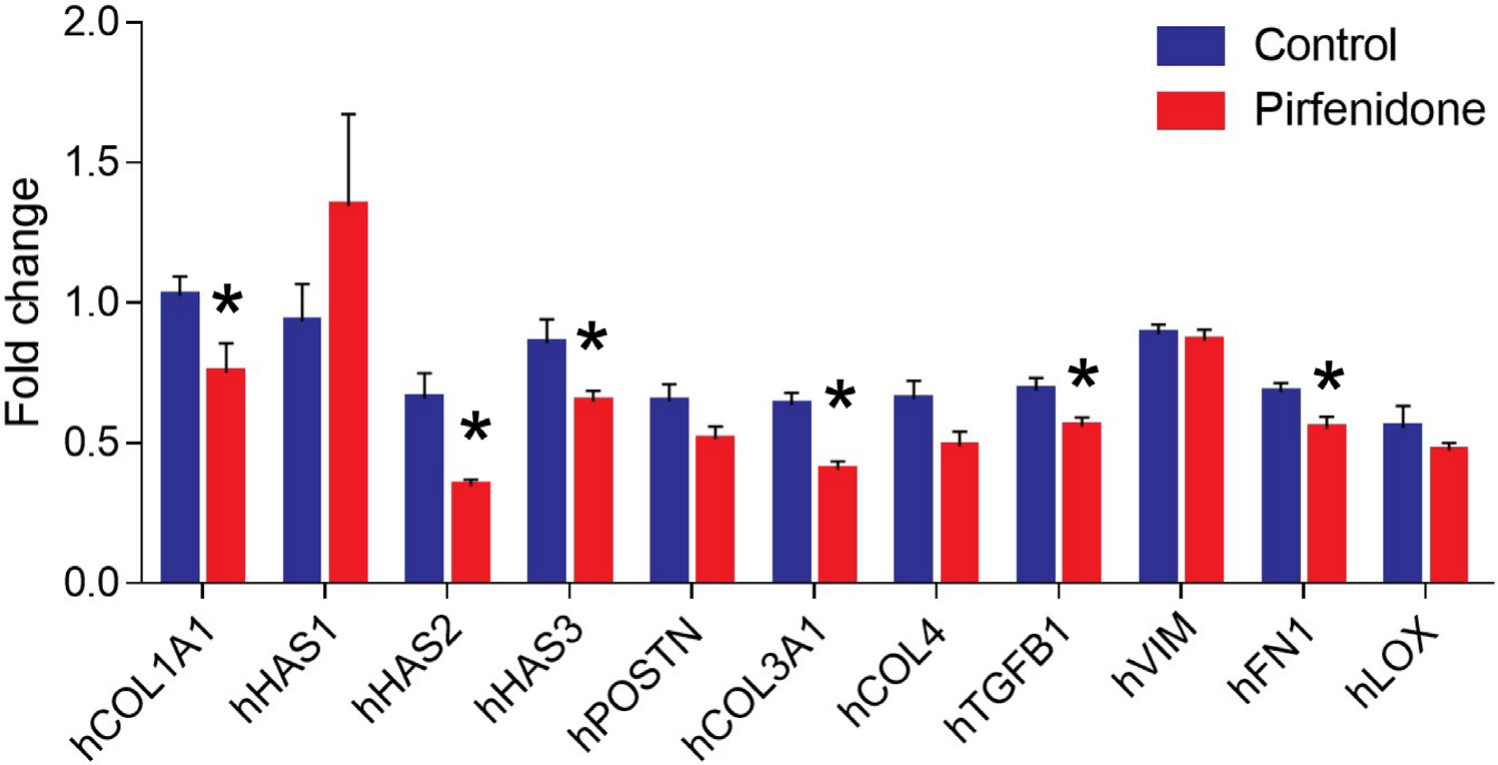
Pirfenidone suppresses human COL1A1, HAS2, HAS3, COL3, TGFB1 and FN1 gene expression levels Real-time PCR gene expression analysis for the quantification of human collagen I, (hCOL1A1), hyaluronan synthase 1 (hHAS1), hyaluronan synthase 2 (hHAS2), hyaluronan synthase 3 (hHAS3), periostin (hPOSTN), collagen 3 (hCOL3A1), collagen 4 (hCOL4A1), transforming growth factor-beta 1 (hTGFβ1), vimentin (hVIM), fibronectin (hFN1), and lysyl oxidase (hLOX) mRNA levels in control-treated compared to Pirfenidone-treated MCF10CA1a tumors. Data indicate that Pirfenidone downregulates expression of COL1A1, HAS2, HAS3, COL3A1, TGFB1 and FN1 genes. Data represent the average expression of each gene normalized to beta-actin from at least 3 independent experiments from 5 control and 5 Pirfenidone-treated tumors. Asterisks indicate statistically significant differences between compared groups (*p* < 0.05).

Based on our histological analysis we concluded that Pirfenidone normalizes the tumor microenvironment by targeting collagen and/or hyaluronan. According to previous studies regarding the Pirfenidone mode of action, we hypothesized that a possible pathway responsible for this effect could be the TGFβ signaling pathway. To elucidate the possible mechanism, we performed real-time PCR gene expression analysis of critical genes that are involved in collagen and hyaluronan synthesis as well as others encoding for extracellular matrix components or collagen crosslinking enzymes. RNA extraction from human MCF10CA1a tumors followed by real-time PCR using human-specific primers showed that Pirfenidone suppressed collagen I (COL1A1), hyaluronan synthase 2 (HAS2), hyaluronan synthase 3 (HAS3), collagen 3 (COL3A1), transforming growth factor-beta 1 (TGFβ1), and fibronectin (FN1) gene expression in breast cancer cells (Figure 6).

## DISCUSSION

Despite the crucial role that mechanical forces play in the delivery of drugs to solid tumors, it was only recently that the contribution of tumor ECM on the development of these forces and the compression of tumor blood vessels was elucidated [5]. Collagen, given its ability to resist to tensile loads, and hyaluronan that swells, owing to its high negative charges, can generate forces large enough to compress fragile intratumoral blood vessels thus, dramatically reducing tumor perfusion. This effect is more evident in desmoplastic tumors, such as breast and pancreatic cancers that are rich in these ECM components. Hypo-perfusion and the resulting reduction in drug delivery might explain, in large part, the low survival rates of patients with these tumor types. In the current study, we explored the hypothesis that normalization of the tumor ECM by repurposing Pirfenidone, an approved anti-fibrotic drug, can alleviate mechanical forces, decompress tumor blood vessels, restore blood vessel functionality and enhance therapeutic outcomes. We confirmed our hypothesis using two orthotopic breast tumor models. Interestingly, in both tumor models chemotherapy alone did not affect tumor growth but when combined with Pirfenidone it caused a significant delay in tumor progression. Recent studies have also shown that Pirfenidone and doxorubicin can synergistically reduce breast tumor progression by targeting tumor fibrosis indirectly via depletion of cancer associated fibroblasts (CAFs) [19]. In our study we demonstrated that targeting the fibrotic ECM will in turn significantly increase blood vessel functionality and perfusion and thus, improve the anti-tumor efficacy of doxorubicin. Moreover, pancreatic cancers are highly desmoplastic and it has been shown that normalization of the tumor microenvironment improves overall survival in preclinical models of pancreatic tumors [2, 20]. Therefore, Pirfenidone could be repurposed to improve chemotherapy in these tumor types. Given the fact that both the anti-fibrotic agent and the cytotoxic drug have been clinically approved, our findings are highly transferable to the clinic and could lead to clinical trials to test the efficacy of this combinatorial treatment in human patients.

Apart from targeting the tumor ECM, normalization of the tumor microenvironment can be also achieved by targeting stromal cells. Inhibition of the Hedgehog cellular signaling pathway with Saridegib to deplete cancer-associated fibroblasts in preclinical pancreatic tumor models has proved to effectively alleviate mechanical forces and improve vessel functionality and efficacy of chemotherapy [5, 21]. This drug, however, recently failed in a phase II clinical trial for pancreatic cancer patients when combined with gemcitabine, presumably due to intrinsic resistance to gemcitabine by these tumors [22]. Furthermore, recent studies have shown that genetic inhibition of Hedgehog pathway accelerates rather than delays tumor progression [23, 24]. We should notice, however, that the mechanisms of action of genetic vs pharmacologic depletion are different as genetic ablation has a greater effect on stromal cell depletion and could potentially destroy instead of normalizing the tumor microenvironment.

Finally, one could argue that opening tumor blood vessels could allow more metastatic cells to disseminate from the primary tumor and increase formation of metastases, which has been shown in a number of pre-clinical studies [25, 26]. We should consider that even though blood vessel functionality is one of the means by which cancer cells are able to metastasize, the creation of a harsh hypoxic microenvironment—owing to hypo-perfusion—is a main cause that forces cancer cells to metastasize. Therefore, restoration of tumor perfusion *via* ECM remodeling can normalize oxygen levels and might reduce the invasive and metastatic potential of cancer cells. Furthermore, drugs that decompress vessels should be given simultaneously with cytotoxic drugs so that improved perfusion to be associated with increased cancer cell killing.

## MATERIALS AND METHODS

### Cell culture

MCF10CA1a human breast cancer cell line was obtained from the Karmanos Cancer Institute (Detroit, MI, USA) and maintained as previously described [27]. 4T1 mouse mammary carcinoma cell line was purchased from ATCC and maintained in RPMI medium supplemented with 10% fetal bovine serum (FBS).

### Drugs and reagents

For *in vivo* studies, Pirfenidone (Esbriet^®^, Roche Pharmaceuticals, Switzerland) was solubilized with sterile water followed by warming at 60^°^C for 30 min [17]. Doxorubicin hydrochloride was purchased as already made solution (2 mg/ml, ACTAVIS) and was solubilized in phosphate buffer saline (PBS).

### Animal tumor models and treatment protocols

Orthotopic xenograft breast tumor models were generated by implantation of 5 × 10^5^ MCF10CA1a cells resuspended in 40 μl of serum-free medium into the mammary fat pad of 6-week old female CD1 nude immunodeficient mice. Orthotopic syngeneic models for murine mammary tumors were generated by implantation of 10^5^ 4T1 mouse mammary cancer cells resuspended in 40 μl of serum-free medium into the mammary fat pad of 6-week old BALB/c female mice. Pirfenidone was administered orally once a day at different doses (350 mg/Kg, 500 mg/Kg or 650 mg/Kg as indicated) from day 4 post-implantation. Doxorubicin (4 mg/kg and 5 mg/kg for the MCF10CA1a and 4T1 models, respectively) was administered by intraperitoneal (i.p.) injection from day 12 post-implantation, every 72 hours. During the course of each experiment, tumor growth was monitored daily and the planar dimensions (*x*, *y*) were measured using a digital caliper. Tumor volume was calculated using the volume of an ellipsoid and assuming that the third dimension, *z*, is equal to $\sqrt{xy}$.All *in vivo* experiments were conducted in accordance with the animal welfare regulations and guidelines of the Republic of Cyprus and the European Union under a license acquired by the Cyprus Veterinary Services (No CY/EXP/PR.L1/2014), the Cyprus national authority for monitoring animal research.

Before the end of each *in vivo* tumor growth experiment, animals were anesthetized by i.p. injection of Avertin (200 mg/kg) and interstitial fluid pressure (IFP) was measure using the wick-in-needle technique [5]. Next, 100 μl biotinylated lectin (1 mg/ml, Vector Labs) was slowly administered in each mouse by intracardiac injection and was allowed to distribute throughout the body for 7 minutes. Finally, mice were sacrificed *via* CO_2_ inhalation and tumors were excised for mechanical, histological and gene expression analysis in order to study modifications in the tumor microenvironment.

### Fluorescent immunohistochemistry and vessel perfusion histology

A detailed description of the staining protocols can be found in the Supplementary Materials. Briefly, transverse 40 μm-thick tumor sections were immunostained with antibodies against collagen I (ab4710, Abcam), CD31 (MEC13.3, BD Biosciences), hyaluronan (ab53842, Abcam), fibronectin (Hybridoma) and a-sma (ab5694, Abcam) counterstained with 4′,6-diamidino-2-phenylindole (Vector Labs). Antigens were detected using appropriate secondary fluorescent antibodies. For blood vessel perfusion analysis, mice were injected with biotinylated lycopersicon esculentum (tomato) lectin (Vector Labs) *via* intracardiac injection prior to euthanization and tumor removal. Streptavidin-conjugated and fluorescently-labelled secondary antibodies against lectin and CD31 were used to detect these antigens, respectively. Images from anti-collagen I, anti-CD31, anti-hyaluronan and anti-biotin-stained sections were analysed based on the area fraction of positive staining. To avoid any bias, the analysis was performed automatically using a previously developed in-house code in MATLAB (MathWorks, Inc., Natick, MA, USA) [5]. Five different sections per tumor (from the interior and the periphery) at ×10 magnification were taken and analyzed.

### Biomechanical analysis

Unconfined compression experiments were carried out for the measurement of the elastic modulus and the hydraulic conductivity of the tumors. For the elastic modulus measurements, tumor specimens 3 × 3 × 2 mm (length × width × thickness) were loaded on a high precision mechanical testing system (Instron, 5944, Norwood, MA, USA) and compressed to a final strain of 30% with a strain rate of 0.05mm/min, the minimum rate the system can apply in order to avoid any transient, poroelastic effects. The modulus was calculated from the slope of the stress-strain curve between 20 and 30% strain. For the calculation of the hydraulic conductivity, stress relaxation experiments were performed in unconfined compression. Specimens underwent four cycles of testing for each of which a 5% compressive strain was applied for 1 minute, followed by a 10 minute hold. Subsequently, a common biphasic model of soft tissue mechanics was employed [28] accounting for both the solid phase (cells and extracellular matrix) and the fluid phase (interstitial fluid) of the tumor. The hydraulic conductivity was calculated by fitting the model to the experimental data.

### Biodistribution analysis

For the biodistribution study, a dose of 9 mg/kg of doxorubicin was administered *via* tail vein injection to the animals 4 hours before sacrificing them. Six animals per group were used (*n* = 6). The tissues were excised from mice and were stored at −80°C until further processing. For doxorubicin dosage and extraction, a previously described protocol was employed [29]. More details can be found in the Supplementary Materials section.

### RNA isolation, cDNA synthesis, and real-time polymerase chain reaction

Total RNA was isolated from breast tumors using standard Trizol-based protocol (Invitrogen) and cDNA synthesis was performed using reverse transcriptase III (RT-III) enzyme and random hexamers (Invitrogen). Real-time polymerase chain reaction (PCR) was performed using Sybr Fast Universal Master Mix (Kapa). The human-specific primers that were used for gene expression analysis are listed in Supplementary Table 1. All reactions were performed using a CFX-96 real-time PCR detection system (Biorad) and the following conditions: 95^°^C for 2 min, 95^°^C for 2 sec, 60^°^C for 20 sec, 60^°^C for 1 sec, steps 2–4 for 39 cycles. Real-time PCR analysis and calculation of changes in gene expression between compared groups was performed using the ΔΔCt method.

### Statistical analysis

The data are presented as means with standard errors. Groups were compared using Student's *t*-test.

## SUPPLEMENTARY MATERIALS FIGURES AND TABLES



## Abbreviations

CD31: cluster of differentiation 31
COL1A1: collagen I
COL3: collagen 3
ECM: extracellular matrix
HAS2: hyaluronan synthase 2
HAS3: hyaluronan synthase 3
IFP: Interstitial fluid pressure
i.p: intraperitoneal
i.v: intravenously
TGFβ1: transforming growth factor-beta 1
