# Supplementary material for: Pirfenidone normalizes the tumor microenvironment to improve chemotherapy
Source: Oncotarget. 2017 Feb 20;8(15):24506–17. doi: 10.18632/oncotarget.15534 (PMC5421866; doi:10.18632/oncotarget.15534)
Supplement: Supplementary file 1 [file oncotarget-08-24506-s001.pdf]

# Pirfenidone normalizes the tumor microenvironment to improve chemotherapy

## Supplementary Materials

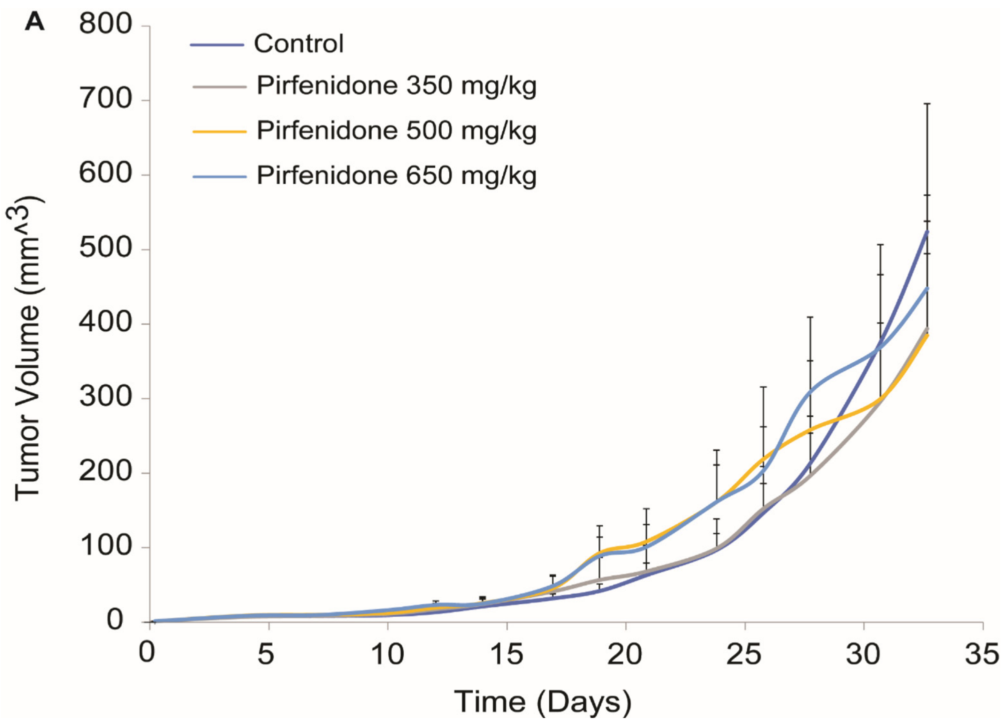


**Supplementary Figure 1: Pirfenidone does not affect tumor volume growth rates.** Tumor volume growth rates of orthotopic MCF10CA1a human breast tumors implanted in female CD1 nude mice. Pirfenidone-treated mice alone show no significantly effect on tumor volume.

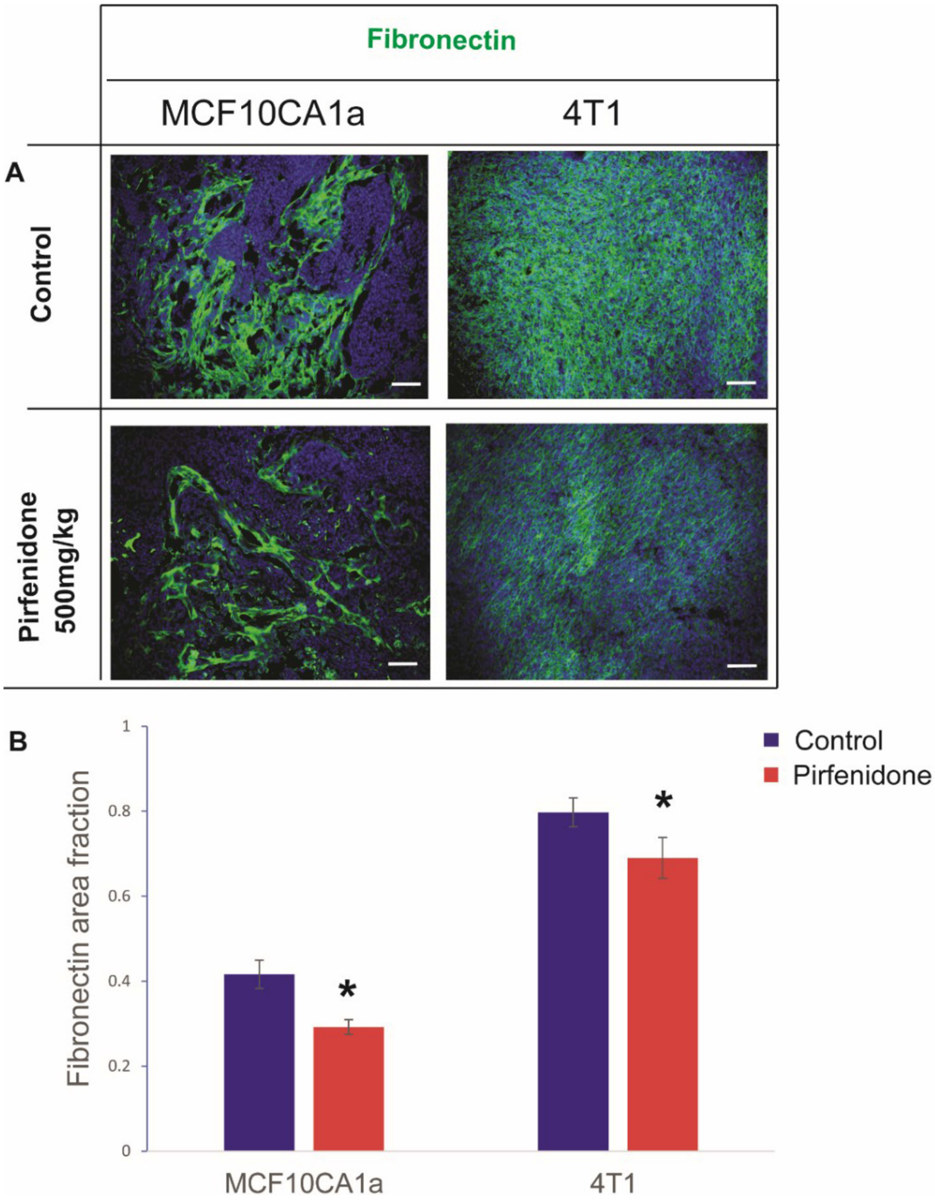


**Supplementary Figure 2: Pirfenidone reduces fibronectin expression in MCF10CA1a and 4T1 breast tumors.** (**A**) Representative immunofluorescent images from histological analysis for MCF10A1a and 4T1 control and Pirfenidone-treated tumors show the effect of Pirfenidone on Fibronectin expression. (**B**) Quantification of area fraction of fibronectin indicates that its expression level is significantly reduced compared to control tumors. Asterisks indicate statistically significant difference between groups ($p < 0.05$). Scale bar: 100 µm.

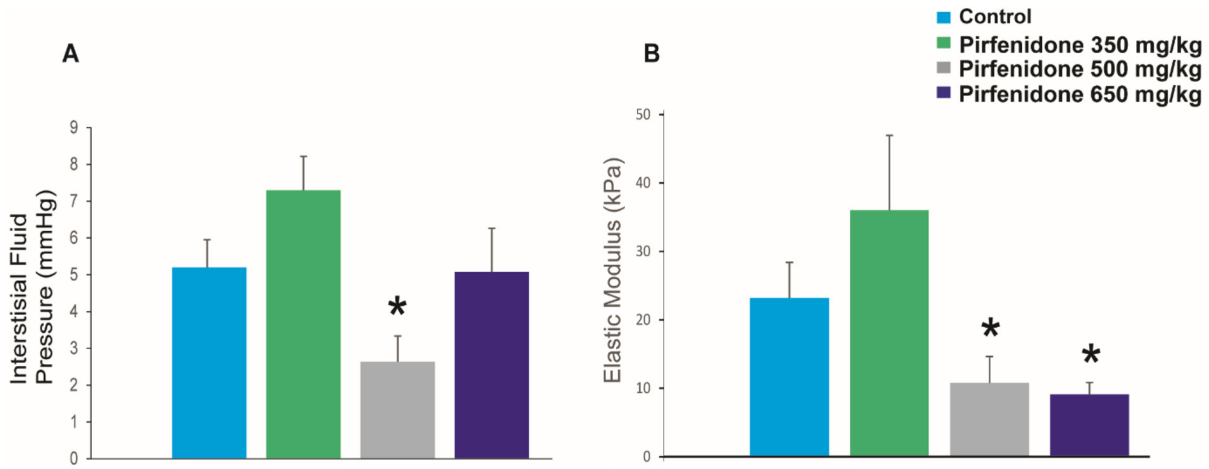


**Supplementary Figure 3: Mechanical properties of Pirfenidone-treated MCF10CA1a breast tumors.** (**A**) The values of the Interstitial Fluid Pressure (IFP) for the Pirfenidone-treated tumors are shown to be significantly lower at 500 mg/kg compared to control as well as to those of 350 mg/kg and 650 mg/kg Pirfenidone-treated MCF10CA1a breast tumors. (**B**) The values of the elastic modulus for the 500 mg/kg and 650 mg/kg Pirfenidone-treated tumors are shown to be significantly lower compared to those of control and 350 mg/kg Pirfenidone-treated MCF10CA1a breast tumors (* indicates $p$ value < 0.05).

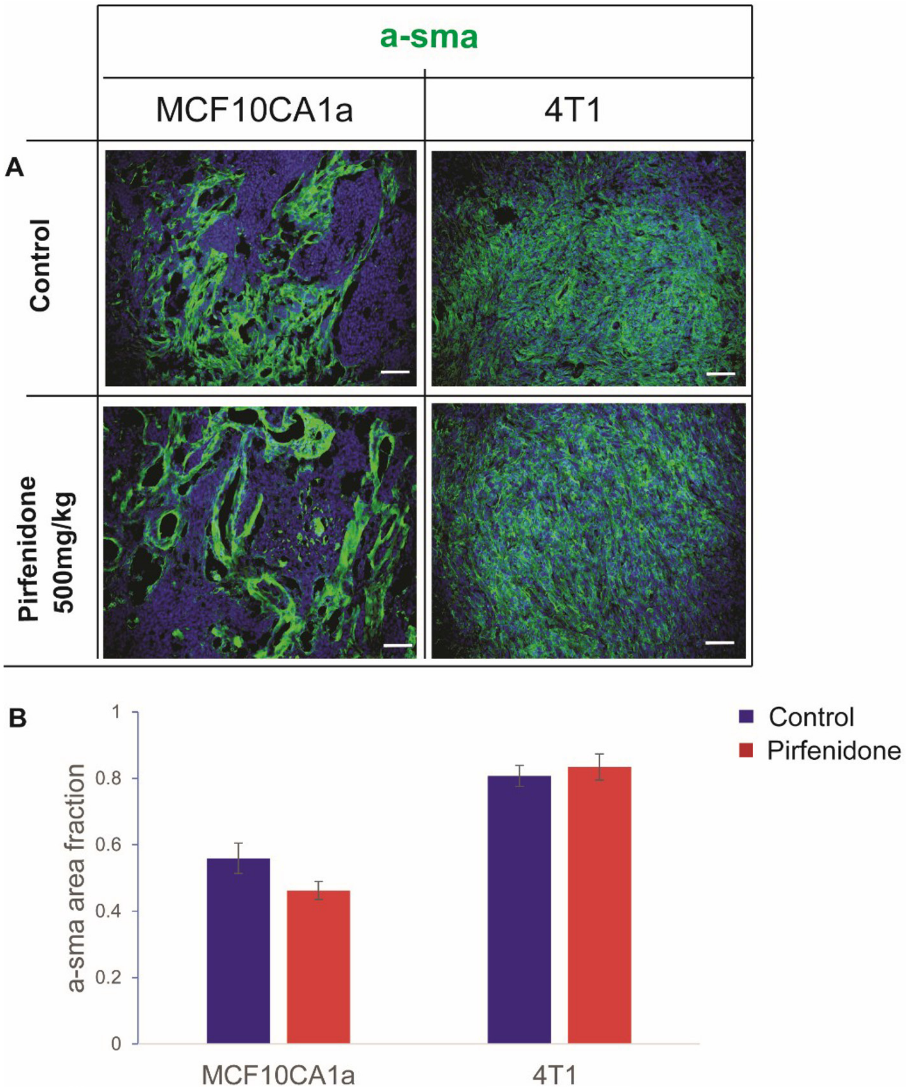


**Supplementary Figure 4: Pirfenidone does not affect alpha smooth muscle actin (a-sma) expression in MCF10CA1a and 4T1 breast tumors.** (**A**) Representative immunofluorescent images of control and Pirfenidone-treated MCF10CA1a and 4T1 tumors for a-sma. (**B**) Area fraction analysis shows no statistically significant difference between compared groups indicating that Pirfenidone has no effect on the expression of a-sma in both breast tumor models. Scale bar: 100 µm.

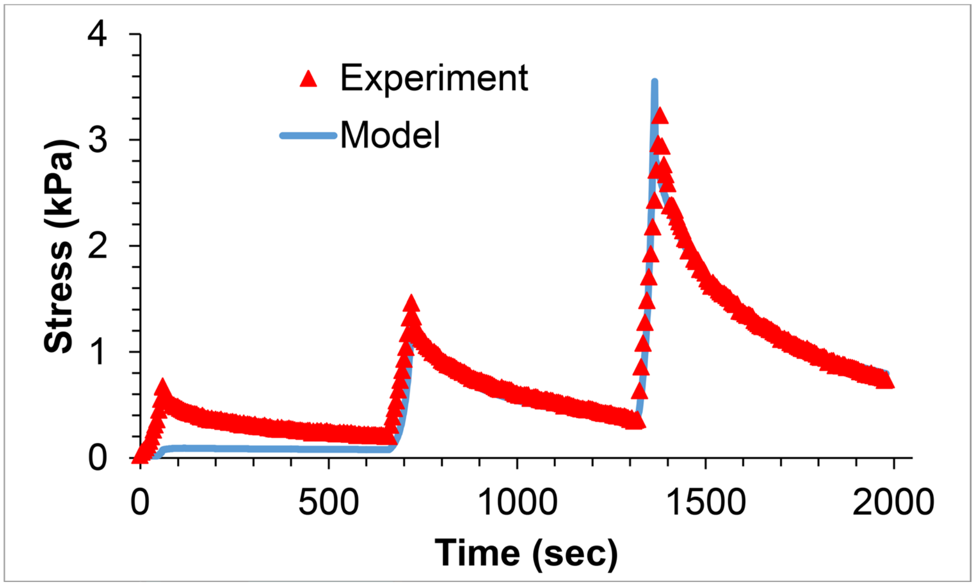


**Supplementary Figure 5: Calculation of the hydraulic conductivity of the tumor.** Representative stress relaxation data of the tumors that were tested along with the fit of a biphasic model [3]. The hydraulic conductivity was calculated by fitting the biphasic model to the experimental data.

**Supplementary Table 1: Human-specific primers that were used for gene expression analysis of cancer cells from MCF10CA1a breast tumors**

| Gene | Primer sequence |
|---|---|
| **hCOL1A1 F** | GTGCTAAAGGTGCCAATGGT |
| **hCOL1A1 R** | ACCAGGTTCACCGCTGTTAC |
| **hHAS1 F** | TCGGAGATTCGGTGGACTAC |
| **hHAS1 R** | GTCCAACCTTGTGTCCGAGT |
| **hHAS2 F** | ACCGGGGTAAAATTTGGAAC |
| **hHAS2 R** | TAAGGCAGCTGGCAAAAGAT |
| **hHAS3 F** | TTTGCCATTGCTACCATCAA |
| **hHAS3 R** | AGGCCAATGAAGTTCACCAC |
| **hB-ACTIN F** | CGAGCACAGAGCCTCGCCTTTGCC |
| **hB-ACTIN R** | TGTCGACGACGAGCGCGGCGATAT |
| **hFN1 F** | GATGCTCCCACTAACCTCCA |
| **hFN1 R** | CGGTCAGTCGGTATCCTGTT |
| **hPOSTN F** | TTCTGACGCCTCAAAACTGA |
| **hPOSTN R** | TGCTCTCCAAACCTCTACGG |
| **hVIM F** | CGAAAACACCCTGCAATCTT |
| **hVIM R** | ATTCCACTTTGCGTTCAAGG |
| **hCOL3 F** | TAGGTCCATCTGGTCCTGCT |
| **hCOL3 R** | CGAAGCCTCTGTGTCCTTTC |
| **hCOL4 F** | CTCTACGTGCAAGGCAATGA |
| **hCOL4 R** | AGAACAGGAAGGGCATTGTG |
| **hLOX F** | CAGAGGAGAGTGGCTGAAGG |
| **hLOX R** | CCAGGACTCAATCCCTGTGT |
| **hTGF-B1 F** | GTACCTGAACCCGTGTTGCT |
| **hTGF-B1 R** | CACGTGCTGCTCCACTTTTA |

# SUPPLEMENTARY MATERIALS AND METHODS

## Pirfenidone normalizes the tumor microenvironment to improve chemotherapy

### Fluorescent immunohistochemistry and vessel perfusion histology

MCF10CA1a and 4T1 breast tumors were excised from mice, fixed and embedded in optimal cutting temperature compound (OCT). Transverse 40 μm-thick tumor sections were produced using the Tissue-Tek Cryo3 (SAKURA) and immunostained with antibodies against collagen I (ab4710, Abcam 1:100 dilution), CD31 (MEC13.3, BD Biosciences, 1:100 dilution) hyaluronan (ab53842, Abcam 1:100 dilution), fibronectin (Hybridoma, 1:50 dilution) and a- smooth muscle actin (ab5694, Abcam, 1:100) counterstained with 4′,6-diamidino-2-phenylindole (Vector Labs). Antigens were detected using appropriate secondary fluorescent antibodies. Collagen I was detected with Alexa Fluor-647 Goat Anti-Rabbit IgG (H+L) secondary antibody (A-21244, Invitrogen, 1:800 dilution), CD31 with Alexa Fluor-647 Goat Anti-Rat IgG (H+L) secondary antibody (A-21247, Invitrogen, 1:800 dilution), and hyaluronan using Alexa Fluor-647 donkey Anti-sheep IgG (H+L) secondary antibody (A-11015, 1:800 dilution). For blood vessel perfusion analysis, mice were slowly injected with 100 μl of 1 mg/ml biotinylated lycopersicon esculentum (tomato) lectin (Vector Labs) *via* intracardiac injection 7 minutes prior to euthanization and tumor removal. Upon excision, tumors were fixed in paraformaldehyde, embedded in OCT and frozen. Transverse 40 μm-thick tumor sections were produced and stained with an antibody against the endothelial marker CD31. Streptavidin-conjugated and fluorescently-labelled secondary antibodies against lectin and CD31 were used to detect these antigens, respectively. Lectin staining was detected using the Streptavidin Alexa Fluor 488 conjugate (Molecular probes, S11223, 1:400 dilution) and CD31 signal was detected with Alexa Fluor-647 Goat Anti-Rat IgG (H+L) secondary antibody (Molecular probes, A-21247, 1:800 dilution). Images from anti-collagen I, anti-CD31, anti-hyaluronan and anti-biotin-stained sections were analysed based on the area fraction of positive staining. To avoid any bias, the analysis was performed automatically using a previously developed in-house code in MATLAB (MathWorks, Inc., Natick, MA, USA) [1]. Vessel diameter was calculated from the CD31 images. Specifically, the procedure of the identification of the non-collapsed vessels was automated in the MATLAB code. The CD31 staining that forms a loop was considered to be a vessel and the short axis of a cross-section fit to an oval was taken. Five different sections per tumor (from the interior and the periphery) at ×10 magnification were taken and analyzed keeping the analysis settings and thresholds the same for all tumors.

### Biodistribution analysis

For the biodistribution study, a dose of 9 mg/kg of doxorubicin was injected via tail vein injection to the animals and 4 hours post injection they were sacrificed. Six animals per group were tested ($n$ = 6). The tissues were excised from the mice and were stored at −80°C until extraction. For doxorubicin extraction, we used a previously described method with modifications [2]. The tissue samples were homogenized by using a homogenizer in 4 parts (v/w) cell lysis buffer (RIPA buffer). Then in 200 μl of the homogenate were added 50 μl 10% Triton X-100 and 750 μl 0.75N hydrochloric acid (HCl) in isopropanol. The mixture was well-vortexed and let overnight at -20oC for doxorubicin extraction. Samples were again vortexed at RT followed by centrifugation for 30 min at 4°C (14000 rpm). The supernatant was then collected from all samples and fluorescence was measured (Ex.: 470 nm, Em: 590 nm) using the Qubit 3.0 Fluorometer (Life Technologies). A standard curve was established by adding known amounts of doxorubicin to homogenates of non-treated tissue samples prior to extraction.

# REFERENCES


1. Stylianopoulos T, Martin JD, Chauhan VP, Jain SR, Diop-Frimpong B, Bardeesy N, Smith BL, Ferrone CR, Hornicek FJ, Boucher Y, Munn LL, Jain RK. Causes, consequences, and remedies for growth-induced solid stress in murine and human tumors. Proc Natl Acad Sci USA. 2012; 109:15101–15108.
2. Laginha KM, Verwoert S, Charrois GJ, Allen TM. Determination of doxorubicin levels in whole tumor and tumor nuclei in murine breast cancer tumors. Clin Cancer Res. 2005; 11:6944–6949.
3. Angeli S, Stylianopoulos T. Biphasic modeling of brain tumor biomechanics and response to radiation treatment. J Biomech. 2016; 49:1524–1531.
